# Effect of morpholine, and 4-methylmorpholine on urethane formation: a computational study

**DOI:** 10.1038/s41598-023-44492-x

**Published:** 2023-10-20

**Authors:** Hadeer Q. Waleed, Rachid Hadjadj, Béla Viskolcz, Béla Fiser

**Affiliations:** 1 Institute of Chemistry, University of Miskolc, Miskolc-Egyetemváros, 3515 Hungary; 2 Higher Education and Industrial Cooperation Centre, University of Miskolc, Miskolc-Egyetemváros, 3515 Hungary; 3 Ferenc Rakoczi II Transcarpathian Hungarian College of Higher Education, Beregszász, Transcarpathia, 90200 Ukraine; 4 Department of Physical Chemistry, Faculty of Chemistry, University of Lodz, Lodz, 90-236 Poland

**Keywords:** Catalysis, Physical chemistry, Polymer chemistry

## Abstract

A theoretical study of urethane formation through the reaction of phenyl isocyanate and butan-1-ol was carried out, without and in the presence of morpholine, and 4-methylmorpholine catalysts. The reaction with and without catalysts was studied at BHandHLYP/6-31G(d) and G3MP2BHandHLYP levels of theories. The reaction mechanism in the presence of catalysts differs significantly from the catalyst-free case and includes seven steps. The catalyst-free system was investigated along with the catalytic process, the geometries were optimized, and the corresponding thermodynamic properties were calculated. Calculated reactant complexes were compared with crystal structures of morpholine, and 4-methylmorpholine complexed with diols found in the literature. The structures were strikingly similar and thus, the validity of the proposed and studied general organocatalytic reaction mechanism was partially verified. Meanwhile, an irregularity in the energy profile occurred due to the zwitterionic nature of an intermediate. To handle the irregularity, a correction was implemented which handles the appearance of a zwitterionic structure and the corresponding energetic properties. The results showed that morpholine is less effective catalyst compared to 4-methylmorpholine, which can be associated with the difference in their PA (1523.95 and 963.07 kJ/mol, respectively). The current results prove the important role of amine catalysts in urethane synthesis which can be applied in polyurethane catalyst design and development.

## Introduction

The first polyurethane (PU) which was capable of competing with nylon was developed by Otto Bayer^1^. This invention is one of the most significant advances in polymer science. Polyurethanes are one of the most versatile and unique polymers utilized in industrial manufacturing^2^. Flexible and rigid foam, paint, coating, adhesive, packaging, insulation, clothing yarn, and synthetic fiber are all PU applications^3–5^. More than two million tonnes of PU is synthesized each year only in the European Union^6^. Polyurethane is a segmented polymer containing soft and hard segments. The flexibility is offered by the soft segments, while the hard segments offer strength^7,8^. PUs are formed by reactions between isocyanate and polyol^9–13^. Catalysts can be considered as one of the most important components of the reaction system besides the starting materials^14^. Amines, inorganic salts, organophosphorus and organometallic catalysts are used for the synthesis of polyurethane^15,16^. However, amine catalysts are the most widely used in the production of polyurethane and its raw materials^17–19^. Especially secondary-amine-containing and tertiary-amine-containing structures are used as catalysts in polyurethane synthesis^20^. Therefore, extensive research there is aimed to understand catalytic PU formation by using both theoretical and kinetic methods^21,22^. Mechanisms of the amine-catalyzed isocyanate–alcohol reactions have been the subject of previous research^20,22–24^. The reaction of phenyl isocyanate (PhNCO) with methanol (MeOH) in acetonitrile, using the computational methods of BHandHLYP/6-31G(d) and G3MP2BHandHLYP, combined with the SMD implicit solvent model was examined without and in the presence of eight different catalysts^20,22^. The result demonstrates the important effect of the studied catalysts on the formation of urethane^20,22^. The catalytic effect of triethylamine on polythiourethane synthesis was also studied and an enhancement in the reaction rate with the increase in the catalyst concentration was experienced^25^. Meanwhile, the reaction of phenyl isocyanate (PhNCO) and stoichiometric butan-1-ol (BuOH) in acetonitrile was also explored in the presence of different aliphatic tertiary amine species by using both experimental and theoretical tools^26^. The computed thermodynamic properties of the reaction are in excellent agreement with the experimentally determined ones. The difference is less than 2 kJ/mol in each catalytic system^26^.

**Figure 1:**
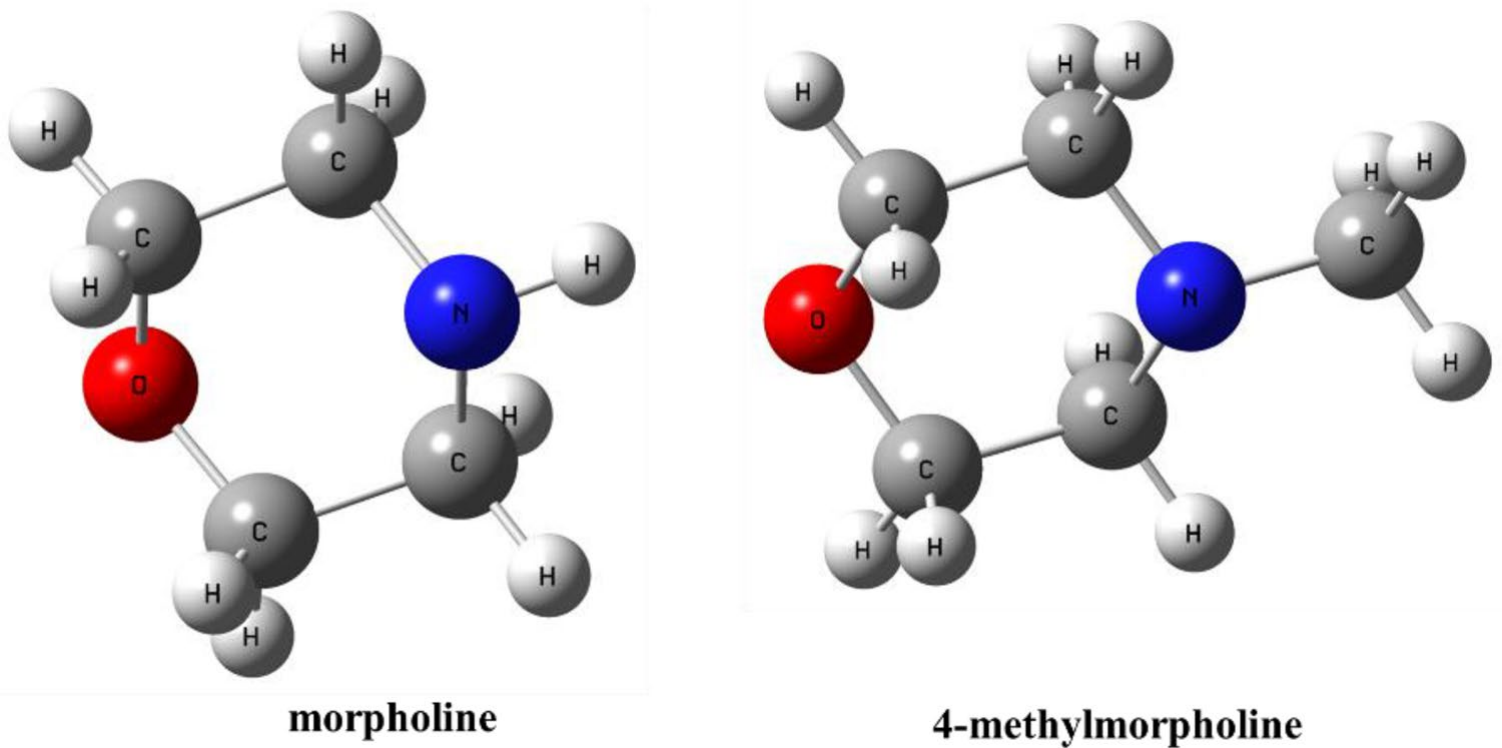
The 3D structures of the studied cyclic amine catalysts.

Although several previous studies were conducted, urethane formation is still a hot topic due to its importance in polyurethane production. The further understanding of the catalytic process is inevitable to achieve more environmentally friendly processes and thus, in this research, the reaction between PhNCO and BuOH is studied in the presence of two different cyclic amine catalysts (Fig. 1). These two cataysts, morpholine, and 4-methylmorpholine are synthetic organic liquids used mainly as an intermediate in the production of rubber chemicals, corrosion inhibitors, waxes and polishes, and optical brighteners. Due to their advantageous physicochemical, biological, and metabolic properties, as well as facile synthetic routes. The morpholine ring is a versatile and readily accessible synthetic building block, it is easily introduced as an amine reagent or can be built according to a variety of available synthetic methodologies^27^.

## Methods

Calculations on the studied systems have been carried out by applying density functional theory and composite methods (BHandHLYP/6-31G(d) and G3MP2BHandHLYP)^28,29^ and using the Gaussian 09 program package^30^. The effect of acetonitrile (MeCN, ε_r_ = 35.688) has also been considered by employing SMD polarizable continuum model to evaluate the effect of the surrounding solvent^31^. Gas phase calculations and other solvents for selected species were also considered using the same solvent model. Furthermore, frequency calculations have also been performed to determine the thermodynamic properties of the species studied and to verify the nature of the stationary points on the potential energy surface. To achieve the G3MP2BHandHLYP energy, geometry optimization, and frequency calculation were performed at the BHandHLYP/6-31G(d) theoretical level. Furthermore, on the optimized structures, two single-point energy calculations were performed on the QCISD(T)/6-31G(d) and MP2/GTMP2Large theoretical levels and the previously determined composite scheme was applied^32^. Meanwhile, an irregularity in the energy profile was identified, where an intermediate (IM) was higher in energy compared to the following transitiona state (TS2). To handle the irregularity in the potential energy surface, a correction of − 24.9 kJ/mol was used which is corresponding to the experimental reaction Gibbs free energy of the appearance of a zwitterionic structure starting from a neutral species in acetonitrile^33,34^. Similar approach was previously applied successfully^33,34^.

## Results and discussion

**Scheme 1:**
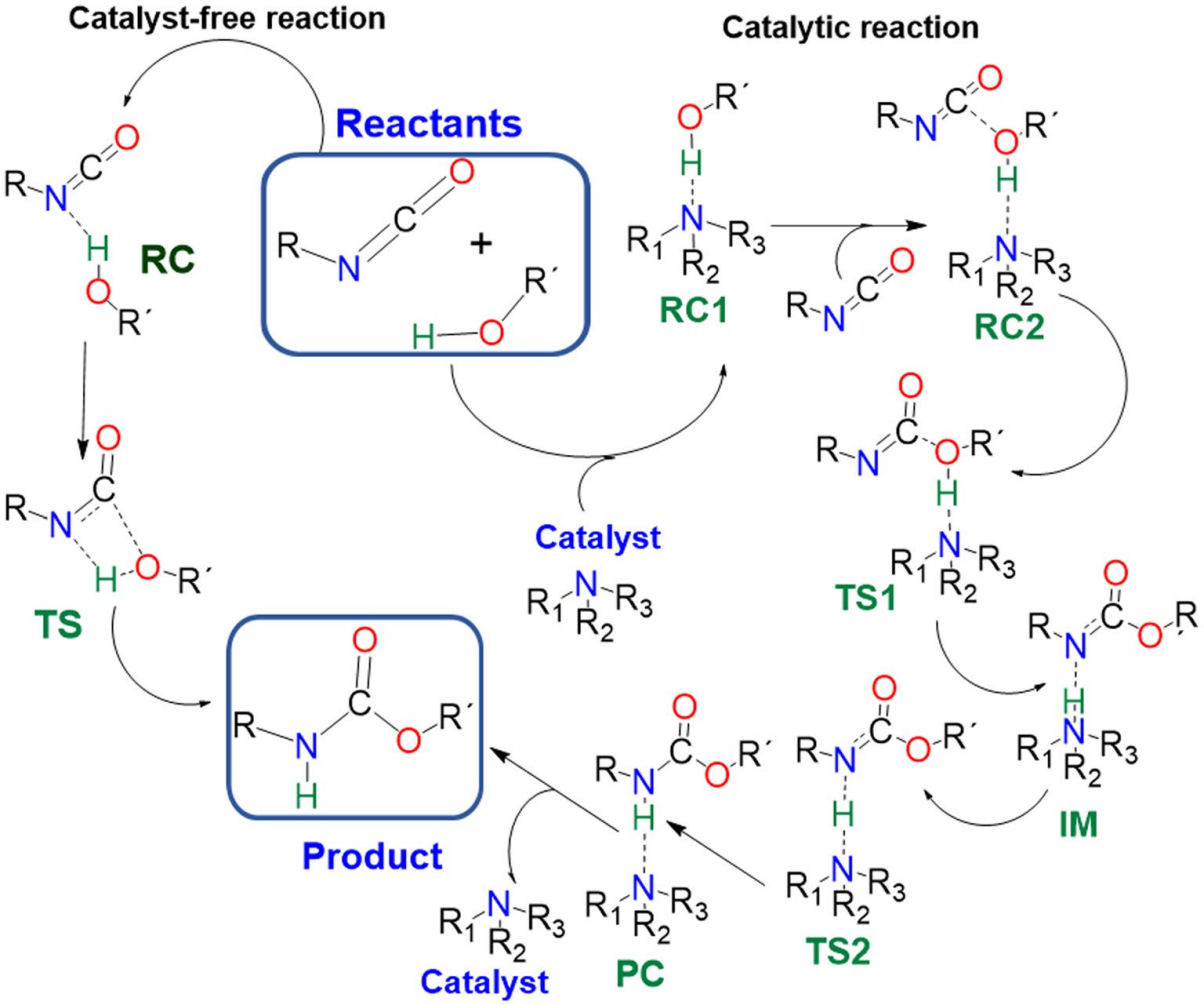
General reaction mechanisms for urethane formation without and in the presence of amine catalysts. RC—reactant complex; TS—transition state; IM—intermediate; PC—product complex.

The formation of the urethane linkage has been studied by using the butan-1-ol and phenyl isocyanate as reference models. Urethane formation without and in the presence of amine catalysts were studied compared by considering the previously proposed general mechanisms^20^ (Scheme 1). The catalyst-free system was investigated along with the catalytic process, the geometries were optimized, and the corresponding thermodynamic properties were calculated, and based on these the reactions were characterized.

The catalyst-free formation of urethane bonds goes through a concerted mechanism (Scheme 1, and Fig. S1) as it was described before. First, the complex will formed (RC, PhNCO–BuOH) and the next step is the formation of the product (P) through a transition state (TS) (Fig. S1).

In the transition state the proton transfer from the hydroxyl group of butan-1-ol to the nitrogen of the isocyanate occurs, and bond is formed between the oxygen of the former and the carbon of the latter. The distances of N–H and C–O within the TS are 1.387 Å and 1.494 Å, respectively. The relative Gibbs free energy which needs to be overcomed in order to obtain the product is 170.05 kJ/mol (Table 1).

**Table 1:** Relative Gibbs free energies (**∆**_**r**_***G***) of the reaction between phenyl isocyanate and butan-1-ol without and in the presence of the studied catalysts, morpholine, or 4-methylmorpholine, calculated at the G3MP2BHandHLYP level of theory in acetonitrile using the SMD implicit solvent model at 298.15 K and 1 atm. The following notations are applied: R—reactants; RC—reactant complex; TS—transition state; IM—intermediate; PC—product complex; P—product.

∆_r_*G* (kJ/mol)								
	R	RC1	RC2	TS1	IM	TS2	PC	P
Cat.-free	0.00	–	28.91*	170.05	–	–	–	− 41.54
morpholine	0.00	15.70	42.45	97.78	12.79 (− 12.11**)	5.46	− 25.18	− 41.54
4-methylmorpholine	0.00	15.05	41.41	97.42	8.77 (− 16.13**)	− 2.68	− 31.56	− 41.54

* RC for catalyst-free (cat.-free) reaction. ** Corrected relative Gibbs free energies calculated according to Ref.^33,34^.

**Figure 2:**
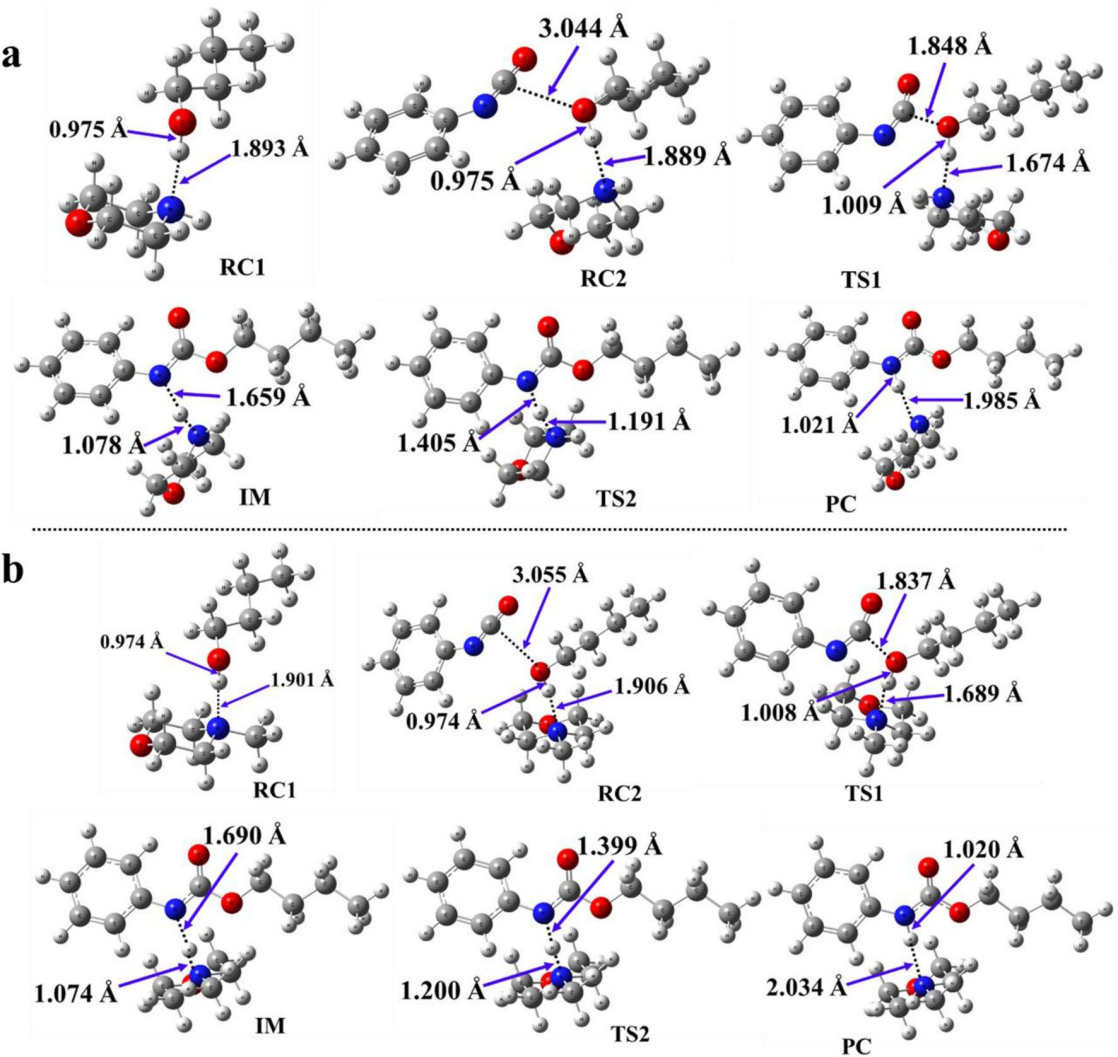
Optimised structures along the reaction pathway between phenyl isocyanate and butan-1-ol in the presence of catalysts: (**a**) morpholine, and (**b**) 4-methylmorpholine calculated at the BHandHLYP/6-31G (d) level of theory in acetonitrile. RC—reactant complex; TS—transition state; IM—intermediate; PC—product complex.

Several additional steps will occur in the case of the mechanism of the phenyl isocyanate – butan-1-ol reaction in the presence of amine catalysts compared to the catalyst-free pathway (Scheme 1) which was proposed before^20^. In this case, the first step will be the formation of the bimolecular reactant complex (RC1), and then a trimolecular complex will emerge (RC2). Hydrogen bond between BuOH and the catalytic amine is formed, and the corresponding N–H* distance is 1.889 Å in case of morpholine and a bit elongated to 1.906 Å when 4-methylmorpholine is considered (Table 2, Fig. 2, and Table S1).

**Table 2:** N–H, O–H, and C–O bond lengths (Å) along the pathway of the phenyl isocyanate (PhNCO) and butan-1-ol reaction in the presence of the studied catalysts, morpholine, and 4-methylmorpholine, calculated at the BHandHLYP/6-31G(d) level of theory in acetonitrile. N–H* for catalysts, while N–H** for PhNCO.

Catalyst	RC1		RC2			TS1			IM		TS2		PC	
	N–H *	O–H	N–H*	O–H	C-O	N–H*	O–H	C-O	N–H*	N–H**	N–H*	N–H**	N–H*	N–H**
Morpholine	1.893	0.975	1.889	0.975	3.044	1.674	1.009	1.848	1.078	1.659	1.191	1.405	1.985	1.021
4-methylmorpholine	1.901	0.974	1.906	0.974	3.055	1.689	1.008	1.837	1.074	1.690	1.200	1.399	2.034	1.020

**Figure 3:**
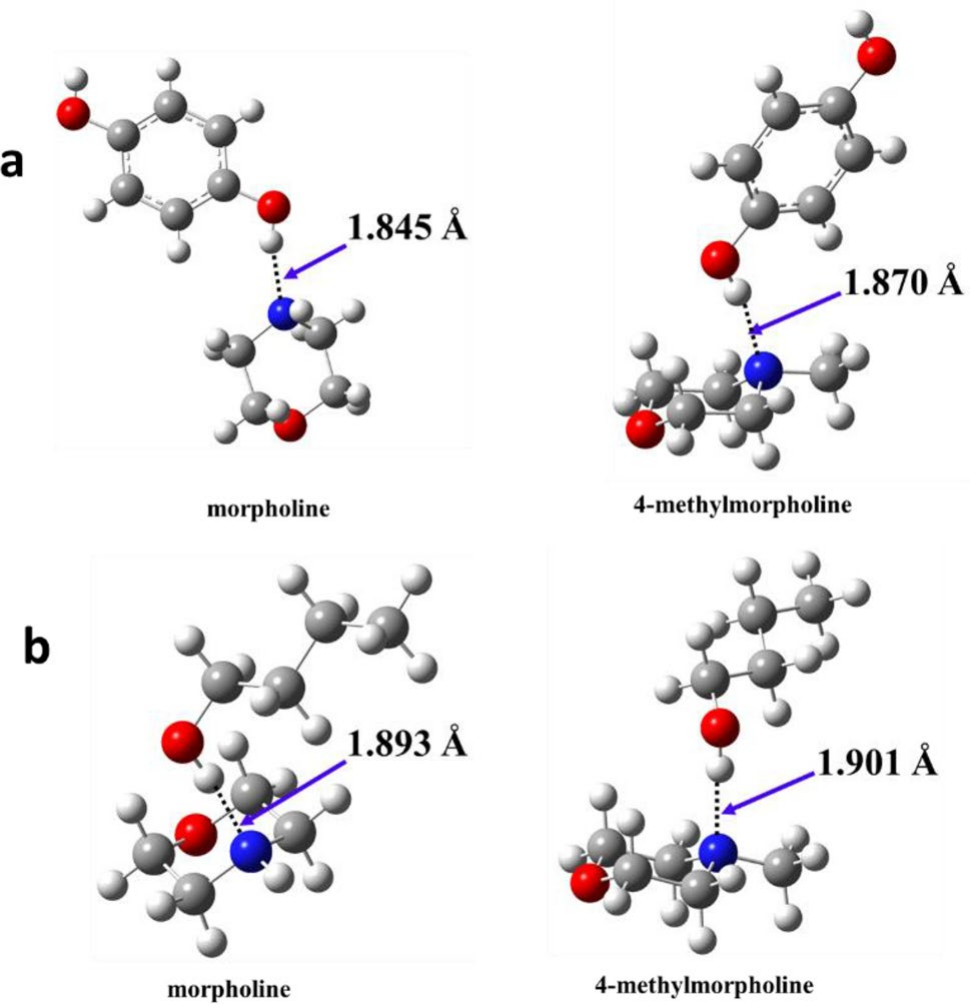
Comparison of morpholine and 4-methylmorpholine complexes: (**a**) crystal structures^37^; (**b**) optimized structures.

To verify the proposed mechanism and the potential formation of the reactant complexes or intermediates, an extensive search for crystal structures in the Cambridge Structural Database (CSD)^36^ was carried out. Crystal structures including morpholine and 4-methylmorpholine complexed with hydroquinone (Fig. 3 “a”) were found^37^. It can be seen that the interaction (−OH–N–, hydrogen bond) between morpholine and the corresponding hydroxyl group in the crystal structure and in the case of the optimized reactant complex (RC1) is very similar to each other and the difference between the length of the hydrogen bonds is only 0.048 Å, while in the case of 4-methylmorpholine it is even smaller 0.031 Å. These promising similarities between the crystal structures and the computed reaction complexes further support the previously proposed complex 7-step mechanism.

Another interaction was established between the carbon of isocyanate and hydroxyl oxygen of BuOH. The corresponding C–O distance is 3.044 Å and 3.055 Å in case of morpholine and 4-methylmorpholine, respectively (Fig. 2). After the reactant complex formation, proton transfer occurs in TS1 from the hydroxyl group to the nitrogen of both morpholine and 4-methylmorpholine, and thus, the corresponding N–H* distances reduced to 1.674 Å and 1.689 Å, respectively (Fig. 2).

**Figure 4:**
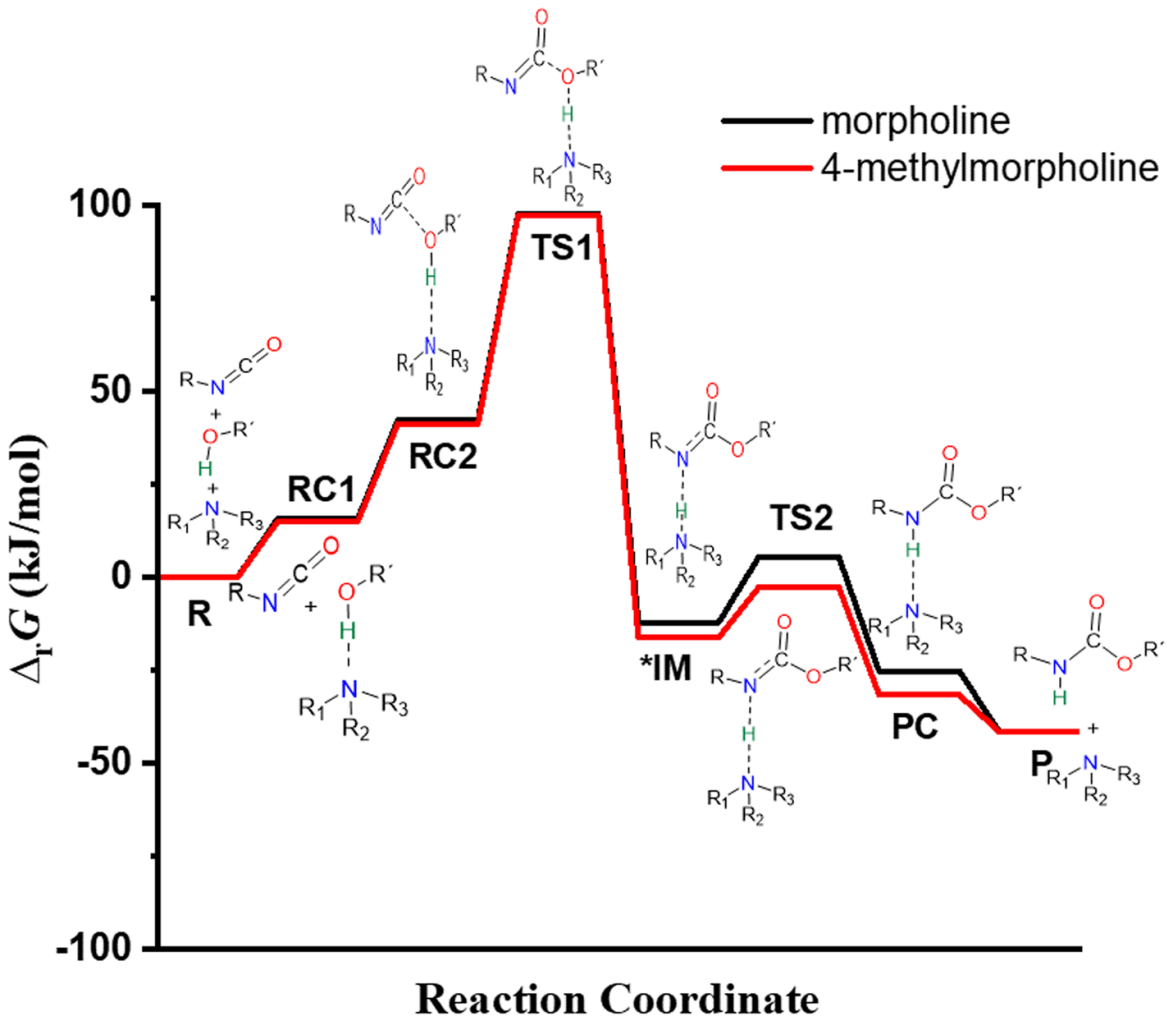
Relative Gibbs free energy (∆_r_*G*) profile of the studied catalysed urethane formation reactions in the presence of morpholine, or 4-methylmorpholine calculated at the G3PMP2BHandHLYP level of theory in acetonitrile using the SMD implicit solvent model at 298.15 K and 1 atm, respectively. *Corrected relative Gibbs free energies of IM calculated according to Ref.^35^.

The relative energy of TS1 is the lowest 97.42 kJ/mol when 4-methylmorpholine is considered, while in case of morpholine a slight increase of ~ 1 kJ/mol is experienced (Fig. 4). However, in both cases the relative energy of the transition state significantly reduced compared to catalyst-free process.

The catalysts are mixed first with the polyol in the experimental preparation steps and thus, RC1 is formed. Thereafter, the trimolecular complex is evolved by adding the isocyanate and the reaction proceeds. Therefore, to compute the barrier height for the first reaction step, the relative energy between TS1 and RC1 has to be computed. In the presence of morpholine and 4-methylmorpholine (Table 1). In previous studies, kinetic experiments of urethane formation were carried out^23,26,38^. The results showed that in the case of cyclic catalysts the activation energy (*E*_a_) cover a wider range between 24.8 and 51.8 kJ/mol, while in the case of linear catalysts it is around 23.9–25.5 kJ/mol. In the current work, the studied catalysts are cyclic structures and the calculated activation energies are 29.7 and 26.6 kJ/mol, for morpholine and 4-methylmorpholine, respectively, which is in good agreement with previous experimental data for similar catalysts. This indicates that morpholine is a bit less effective to prepare urethane compared to its methylated counterpart. A zwitterionic intermediate structure (IM) will form after TS1 within which a new bond will develop between the isocyanate’s carbon and butan-1-ol. The corresponding relative Gibbs free energies of the IMs are 12.79 and 8.77 kJ/mol, for the morpholine and 4-methylmorpholine catalyzed reaction, respectively (Table 1).

The second transition state (TS2) will form where the proton transfer from the catalyst to the product occurs. In TS2, the distance between N–H** decreased, while the distance between N–H* increased compared to IM (Table 2, Fig. 2), and the relative energies are differed by ~ 10 kJ/mol in case of morpholine and 4-methylmorpholine. The TS2 structures including morpholine and 4-methylmorpholine have a relative energy of 5.46 kJ/mol and − 2.68 kJ/mol, respectively.

It seems that IM is higher in energy than TS2, ΔΔ_r_G__TS2-IM_ = − 7.32 kJ/mol and − 11.45 kJ/mol in case of morpholine and 4-methylmorpholine, respectively (Table 1), which needs to be explained. The most straightforward explanation is the solvent effect, which is caused by the zwitterionic nature of the intermediate. Despite numerous attempts, IM was not located in the gas phase, which is also related to its zwitterionic nature. Furthermore, calculations were also carried out in different solvents and it was found that by changing solvent the relative energy of TS2 and IM is also changing (Table S3) similarly in the literature^35^, but the IM remained higher than the corresponding transition state. Thus, the applied method is not suitable to handle the solvent effect precisely in case of the zwitterionic intermediate. Therefore, a correction was applied which was previously successfully used in the literature to handle a system within which zwitterionic structure is formed in case of an amino acid^34^. To balance the effect of the formation of the zwitterionic IM, − 24.9 kJ/mol^33^ was added to the relative Gibbs free energy which shifted the uncorrected ΔΔ_r_G__TS2-IM_ from − 7.32 kJ/mol and − 11.45 kJ/mol to 17.58 and 13.45 kJ/mol in case of the morpholine and 4-methylmorpholine catalyzed process, respectively. By applying the correction, the IM became lower in energy than TS2, and thus, the previous irregularity in the energy profile has been handled.

Since the catalytic mechanism includes proton transfers proton affinities (PAs) for the active nitrogens of the catalysts are also calculated (Fig. 1, Table 3). It was found that morpholine is better proton acceptor, as it had higher proton affinity (1523.95 kJ/mol). While, 4-methylmorpholine has a lower proton affinity (963.07 kJ/mol), after protonation, it is more prone to donate the proton.

**Table 3:** Computed (PA_calc_) of the amines of the studied catalysts, morpholine, and 4-methylmorpholine, in kJ/mol. The calculations were carried by using the G3MP2BHandHLYP composite method in the gas phase at 298.15 K and 1 atm.

Catalysts	PA_calc_ (kJ/mol)
Morpholine	1523.95
4-methylmorpholine	963.07

The formation of the product complex (PC) is the penultimate step of the reaction, it is a bimolecular compound of the catalyst and product (Fig. 2). In the last step, the catalyst is separated from the product. The reaction is significantly changed in the presence of catalysts compared to the case of catalyst-free system (Fig. 4). The product forms in multiple steps and the relative energy is significantly reduced in case of the organocatalytic reaction. The proton affinity of the catalytic site affects the relative energy of the reaction steps (e.g., TS1, IM, TS2) and by increasing proton affinity the corresponding relative energy is also increasing and thus, the lower proton affinitiy can be associated with better catalytic effect. Considering the studied catalysts, 4-methylmorpholine is more effective to promote urethane formation than morpholine.

## Conclusions

The reaction mechanism of the urethane formation from phenyl isocyanate and butan-1-ol without and in the presence of morpholine and 4-methylmorpholine catalysts was determined using computational chemistry tools including both density functional theory (BHandHLYP/6-31G(d)) and composite (G3MP2BHandHLYP) methods. The proposed mechanism for urethane formation in the presence of morpholine or 4-methylmorpholine catalyst contains seven steps compared to the catalyst-free reaction mechanism. To verify the proposed mechanism crystal structures of morpholine, and 4-methylmorpholine complexed with diols found in the literature were compared with calculated reactant complexes. Based on the structural similarity the proposed and studied general organocatalytic reaction mechanism was partially verified. In terms of the catalytic activity, morpholine is a bit less effective to promote urethane formation compared to its methylated counterpart. The relative energy of the TS1 is lower (97.42 kJ/mol) when 4-methylmorpholine is considered, while in the case of morpholine an increase of ~ 1 kJ/mol is experienced. However, in both cases, the relative energy of the transition state was significantly reduced compared to the catalyst-free process. An irregularity in the energy profiles was found, where the IM is higher in energy compared to the TS2, ∆∆_r_G__TS2-IM_ = − 7.32 kJ/mol and − 11.45 kJ/mol in the case of morpholine and 4-methylmorpholine catalyzed process, respectively. This irregularity was successfully explained and a correction was applied to balance the effect of the formation of the zwitterionic IM, which shifted the uncorrected difference (∆∆_r_G__TS2-IM_) to 17.58 and 13.45 kJ/mol for the morpholine and 4-methylmorpholine containing system, respectively. By applying the correction, the IM is lower in energy than TS2, and thus, the irregularity in the energy profile has been handled. Meanwhile, as the proposed catalytic mechanism contains protonation steps, therefore the corresponding PA values (1523.95 and 963.07 kJ/mol, for morpholine and 4-methyl morpholine, respectively) have been calculated. The PA of the catalytic site affects the relative energy of the reaction steps (e.g., TS1, IM, TS2) and by increasing proton affinity the corresponding relative energy is also increasing and thus, the lower proton affinity can be associated with better catalytic effect. Based on the results it was found that the studied cyclic catalysts can be effectively applied in organocatalytic urethane synthesis.

### Supplementary Information

Supplementary Information.
