# Supplementary material for: Effect of morpholine, and 4-methylmorpholine on urethane formation: a computational study
Source: Sci Rep. 2023 Oct 20;13:17950. doi: 10.1038/s41598-023-44492-x (PMC10589236; doi:10.1038/s41598-023-44492-x)
Supplement: Supplementary file 1 — Supplementary Information. [file 41598_2023_44492_MOESM1_ESM.docx]

**Effect of morpholine, and 4-methylmorpholine on urethane formation: a computational study**

**Hadeer Q. Waleed ^1,2^, Rachid Hadjadj^1,2^, Béla Viskolcz ^1,2^, and Béla Fiser ^2,3,4,*^**

^1^ Institute of Chemistry, University of Miskolc, 3515 Miskolc-Egyetemváros, Hungary;

^2^ Higher Education and Industrial Cooperation Centre, University of Miskolc, 3515 Miskolc-Egyetemváros,Hungary

^3^ Ferenc Rakoczi II Transcarpathian Hungarian College of Higher Education, 90200 Beregszász, Transcarpathia, 90200, Ukraine

^4^ Department of Physical Chemistry, Faculty of Chemistry, University of Lodz, Lodz, 90-236, Poland

**^*^** Correspondence: bela.fiser@uni-miskolc.hu (B.F.)

**Supporting Information**


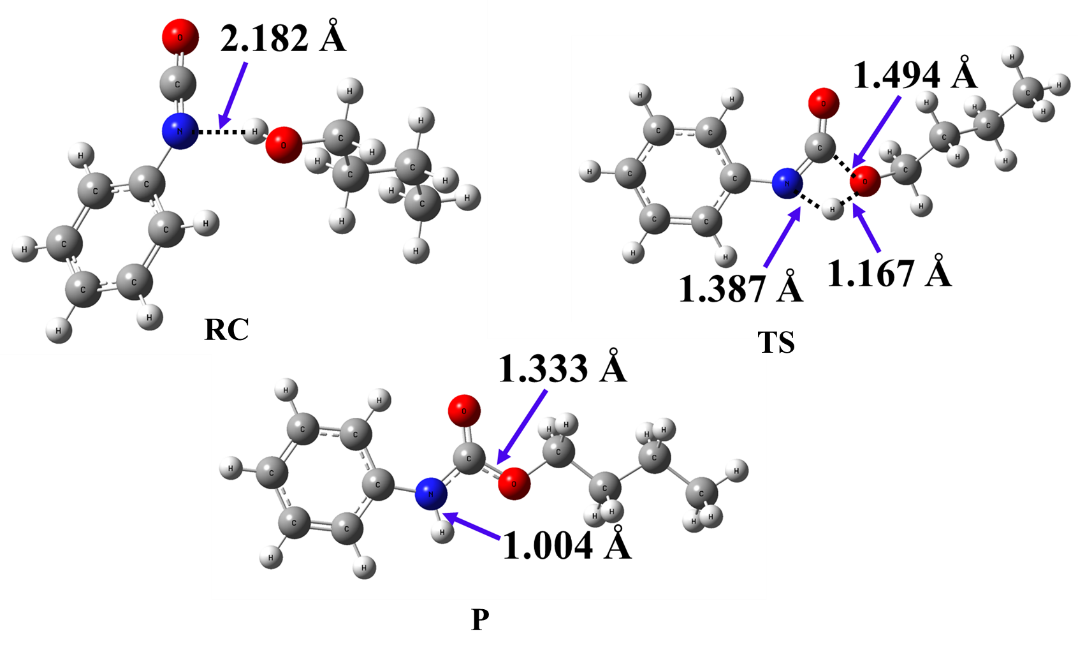


**Figure S1.** Optimised structures along the reaction pathway between phenyl isocyanate and butan-1-ol calculated at the BHandHLYP/6-31G(d) level of theory in acetonitrile. RC—reactant complex; TS—transition state; P—product.

**Figure S2.** Relative enthalpy (∆_r_*H*) profile of the studied catalysed urethane formation reactions in the presence of morpholine, or 4-methylmorpholine calculated at the G3PMP2BHandHLYP level of theory in acetonitrile using the SMD implicit solvent model at 298.15 K and 1 atm, respectively.

**Table S1.** Zero-point corrected relative energies (∆_r_*E*_0_), enthalpies (∆_r_*H*), Gibbs free energies (∆_r_*G*), and entropies (∆_r_*S*) of the reaction between phenyl isocyanate and butan-1-ol in the presence morpholine, or 4-methylmorpholine calculated at the BHandHLYP/6-31G(d) level of theory in acetonitrile using the SMD implicit solvent model at 298.15 K and 1 atm. R—reactant; RC—reactant complex; TS—transition state; IM—intermediate; PC—product complex; P—product.

|  | ∆_r_*E*_0_ (kJ/mol) | | | | | | | | |
| --- | --- | --- | --- | --- | --- | --- | --- | --- | --- |
|  | **R** | | **RC1** | **RC2** | **TS1** | **IM** | **TS2** | **PC** | **P** |
| Cat.-free | 0.00 | | - | -10.48* | 122.75 | - | - | - | -120.94 |
| morpholine | 0.00 | | -26.09 | -29.69 | 10.63 | -89.70 | -97.67 | -140.75 | -120.94 |
| 4-methylmorpholine | 0.00 | | -24.18 | -28.31 | 15.43 | -85.03 | -93.13 | -138.45 | -120.94 |
| ∆_r_*H* (kJ/mol) | | | | | | | | | |
|  | **R** | **RC1** | | **RC2** | **TS1** | **IM** | **TS2** | **PC** | **P** |
| Cat.-free | 0.00 | - | | -8.23* | 120.13 | - | - | - | -123.20 |
| morpholine | 0.00 | -24.73 | | -24.73 | 10.52 | -99.75** | -99.58 | -140.76 | -123.20 |
| 4-methylmorpholine | 0.00 | -22.79 | | -23.34 | 14.91 | -95.22** | -94.91 | -138.40 | -123.20 |
| ∆_r_*G* (kJ/mol) | | | | | | | | | |
|  | **R** | | **RC1** | **RC2** | **TS1** | **IM** | **TS2** | **PC** | **P** |
| Cat.-free | 0.00 | | - | 29.65* | 173.69 | - | - | - | -69.89 |
| morpholine | 0.00 | | 13.92 | 49.13 | 102.99 | -15.43 ** | 3.32 | -45.47 | -69.89 |
| 4-methylmorpholine | 0.00 | | 17.47 | 52.96 | 112.91 | -8.09 ** | 8.36 | -42.60 | -69.89 |
| ∆_r_*S* (cal/mol*K) | | | | | | | | | |
|  | **R** | | **RC1** | **RC2** | **TS1** | **IM** | **TS2** | **PC** | **P** |
| Cat.-free | 0.00 | | - | -30.4* | -97.3 | - | - | - | -133.4 |
| morpholine | 0.00 | | -30.99 | -59.21 | -74.12 | -80.26 | -82.49 | -76.39 | -133.4 |
| 4-methylmorpholine | 0.00 | | -32.27 | -61.16 | -78.56 | -82.51 | -82.79 | -76.80 | -133.4 |

* RC for catalyst-free (cat.-free) reaction. ** Corrected values calculated according to ref. ^34^.

**Table S2.** Relative Gibbs free energies (∆_r_*G*), Zero-point corrected relative energies (∆_r_*E*_0_), enthalpies (∆_r_*H*), and entropies (∆_r_*S*) of the reaction between phenyl isocyanate and butan-1-ol in the presence morpholine, or 4-methylmorpholine calculated at the G3MP2BHandHLYP level of theory in acetonitrile using the SMD implicit solvent model at 298.15 K and 1 atm. R—reactant; RC—reactant complex; TS—transition state; IM—intermediate; PC—product complex; P—product.

| ∆_r_*E*_0_ (kJ/mol) | | | | | | | | | |
| --- | --- | --- | --- | --- | --- | --- | --- | --- | --- |
|  | **R** | **RC1** | | **RC2** | **TS1** | **IM** | **TS2** | **PC** | **P** |
| Cat.-free | 0.00 | - | | -11.22* | 119.11 | - | - | - | -92.58 |
| morpholine | 0.00 | -24.32 | | -36.37 | 5.42 | -86.39 | -95.53 | -120.47 | -92.58 |
| 4-methylmorpholine | 0.00 | -26.61 | | -39.87 | -0.06 | -93.08 | -104.17 | -127.42 | -92.58 |
| ∆_r_*H* (kJ/mol) | | | | | | | | | |
|  | **R** | | **RC1** | **RC2** | **TS1** | **IM** | **TS2** | **PC** | **P** |
| Cat.-free | 0.00 | | - | -8.97* | 116.49 | - | - | - | -94.84 |
| morpholine | 0.00 | | -22.95 | -31.41 | 5.31 | -125.44** | -97.44 | -120.47 | -94.84 |
| 4-methylmorpholine | 0.00 | | -25.21 | -34.89 | -0.58 | -132.26** | -105.96 | -127.37 | -94.84 |
| ∆_r_*S* (kJ /mol*K) | | | | | | | | | |
|  | **R** | | **RC1** | **RC2** | **TS1** | **IM** | **TS2** | **PC** | **P** |
| Cat.-free | 0.00 | | - | -0.13* | -0.41 | - | - | - | -0.56 |
| morpholine | 0.00 | | -0.13 | -0.25 | -0.31 | -0.34 | -0.35 | -0.32 | -0.56 |
| 4-methylmorpholine | 0.00 | | -0.14 | -0.26 | -0.33 | -0.35 | -0.35 | -0.32 | -0.56 |

* RC for catalyst-free (cat.-free) reaction. ** Corrected values calculated according to ref. ^34^.

**Table S3.** Relative Gibbs free energy (∆Δ_r_*G*__TS2-IM_) for intermediate (IM) and TS2 in various media.

| Medium | Dielectric constant (ε) |  | ∆Δ_r_*G* (kJ/mol) |
| --- | --- | --- | --- |
|  |  | **morpholine** | **4-methylmorpholine** |
| Water | 80 | -4.79 | -5.98 |
| Acetonitrile | 37.5 | -6.15 | -8.45 |
| Tetrahydrofuran | 7.58 | -4.03 | -6.03 |

**Table S3.** Cartesian coordinates of the stationary points for the reaction system studied. The calculated at the BHandHLYP/6-31G(d) level of theory in acetonitrile at 298.15 K and 1 atm. Cat.- catalysts, RC – reactant complex, TS – transition state, IM – intermediate, PC – product complex, P – product.

| **morpholine** | **morpholine -RC1** |
| --- | --- |
| **C 0.00608900 -0.77581900 1.16735800**  **H -1.02197000 -1.14337900 1.21743800**  **H 0.55866200 -1.17819400 2.01077500**  **C 0.00608900 -0.77581900 -1.16735800**  **H 0.55866200 -1.17819400 -2.01077500**  **H -1.02197000 -1.14337900 -1.21743800**  **C 0.00608900 0.73877200 -1.19526600**  **H -0.52445900 1.08824400 -2.07781000**  **H 1.04269900 1.08804300 -1.26371200**  **C 0.00608900 0.73877200 1.19526600**  **H -0.52445900 1.08824400 2.07781000**  **H 1.04269900 1.08804300 1.26371200**  **N -0.66485200 1.22775600 0.00000000**  **O 0.63271300 -1.26190600 0.00000000**  **H -0.66373100 2.23608300 0.00000000** | **O -0.47905700 -1.68614600 -0.07121900**  **N 1.31323200 0.21000500 1.11434700**  **C 2.56561100 -0.53804400 1.17978300**  **H 3.33433700 0.00809600 1.73393300**  **H 2.39130100 -1.48521300 1.68343100**  **C 1.50447700 1.46901800 0.40060400**  **H 2.21470300 2.12048600 0.91798100**  **H 0.55425800 1.99148800 0.33010100**  **C -1.53523200 -1.04591400 -0.73929300**  **H -1.15929600 -0.28780800 -1.43322300**  **H -2.03191700 -1.80400200 -1.34356200**  **H 0.08028700 -1.00137800 0.34180400**  **C 2.03251800 1.17078200 -0.98759900**  **H 2.25801600 2.09135700 -1.51656900**  **H 1.28206700 0.61821400 -1.55817600**  **C 3.05416200 -0.78665100 -0.23218900**  **H 2.33533400 -1.41672900 -0.76066400**  **H 4.01674700 -1.28789000 -0.22402700**  **C -2.54022700 -0.41250900 0.20890400**  **H -2.02828900 0.33268000 0.82077400**  **H -2.90377600 -1.18054300 0.89259200**  **C -3.71314500 0.23816100 -0.51259900**  **H -4.22208600 -0.51106700 -1.12024200**  **H -3.33784000 0.99074600 -1.20728000**  **C -4.71215700 0.88244300 0.43719600**  **H -4.23939400 1.66038900 1.03497100**  **H -5.53838300 1.33802700 -0.10527200**  **H -5.13130900 0.14835700 1.12379400**  **O 3.23082600 0.42901700 -0.92735400**  **H 0.98243600 0.39808100 2.04894600** |
| **morpholine -RC2** | **morpholine -TS1** |
| **O 1.37286900 -0.51131800 -1.30722900**  **C -1.01171100 -2.07733400 -0.24302900**  **N 1.88643300 2.28564300 -1.02244700**  **C 0.54307200 2.85789400 -0.99533800**  **H 0.56243900 3.94148800 -1.14268100**  **H -0.04898400 2.41659600 -1.79287400**  **C 2.69357200 2.81443300 0.07290300**  **H 2.83342000 3.89559300 -0.01646400**  **H 3.67301400 2.34394300 0.05806300**  **C 2.54736000 -1.27956800 -1.33490000**  **H 2.27592800 -2.25375100 -1.73741300**  **C -5.69654700 -0.05631600 0.39752300**  **C -5.22232700 -1.28293100 0.84193800**  **C -4.85427600 0.78935600 -0.30894700**  **C -3.54599100 0.41628600 -0.57182200**  **C -3.07730100 -0.81233800 -0.12456400**  **C -3.91700100 -1.66604800 0.58504600**  **H -3.54456200 -2.61722400 0.92973300**  **H -5.86966300 -1.94687200 1.39182200**  **H -5.21372700 1.74363300 -0.65848300**  **H -2.88446800 1.06487200 -1.12168200**  **N -1.75410800 -1.15372500 -0.40596800**  **O -0.23030400 -2.93348100 -0.13069800**  **H 1.61040900 0.41989700 -1.13856200**  **H 3.27993300 -0.84577200 -2.02279500**  **H -6.71366300 0.23669600 0.60047700**  **C 1.99818600 2.51630000 1.38500800**  **H 2.53803300 2.96365300 2.21358800**  **H 1.95225000 1.43628900 1.54340600**  **C -0.08982200 2.55226700 0.34655600**  **H -0.21253100 1.47244300 0.45823900**  **H -1.06516600 3.02152000 0.42663400**  **C 3.17963900 -1.45687400 0.03554100**  **H 3.44363000 -0.47614700 0.43584600**  **H 2.43089800 -1.87894700 0.70590000**  **C 4.41603800 -2.34618600 0.01109800**  **H 4.14808600 -3.32340200 -0.39259000**  **H 5.15233200 -1.92441300 -0.67431300**  **C 5.04744500 -2.52317300 1.38424300**  **H 4.34457300 -2.97305300 2.08393900**  **H 5.92543000 -3.16470400 1.33826800**  **H 5.35828800 -1.56566400 1.79980300**  **O 0.69590500 3.05843000 1.40370000**  **H 2.32804900 2.50623800 -1.90267200** | **O 0.70211800 -0.89135500 -0.24169100**  **C -1.02541200 -1.34848800 0.22995800**  **N 0.73488000 1.77834300 -0.46269100**  **C 0.56372800 2.49213600 0.80301800**  **H 0.19767100 3.50697100 0.63338900**  **H -0.16434600 1.96272900 1.41107800**  **C 1.76554600 2.41033100 -1.28564500**  **H 1.47055600 3.42098800 -1.57585900**  **H 1.91033200 1.82351800 -2.18842900**  **C 1.78587900 -1.47600900 0.46890000**  **H 2.13781100 -0.77928600 1.22753300**  **H 1.38500600 -2.34790500 0.97543200**  **C -5.84407100 -0.07223100 -0.12119700**  **C -5.23201300 -1.17666300 0.45284800**  **C -5.05805600 0.89707100 -0.72867400**  **C -3.68072900 0.76232400 -0.76069500**  **C -3.05948200 -0.34550400 -0.18496200**  **C -3.85338600 -1.31864900 0.42501200**  **H -3.39594700 -2.18307000 0.87549100**  **H -5.82959300 -1.93847900 0.92832400**  **H -5.51755300 1.76199700 -1.18037000**  **H -3.06631000 1.51205600 -1.23265800**  **N -1.65678200 -0.39411000 -0.27032400**  **O -0.93967900 -2.36830000 0.81708800**  **H 0.78605700 0.11241900 -0.30534300**  **H -6.91674100 0.03149600 -0.09591500**  **C 3.05704300 2.47827100 -0.49795400**  **H 3.81570600 3.01383600 -1.05938400**  **H 3.42416200 1.46881100 -0.29678000**  **C 1.89473500 2.55988400 1.52173800**  **H 2.21929000 1.55467100 1.80169000**  **H 1.80827800 3.15653400 2.42404800**  **C 2.90441600 -1.87141500 -0.47304400**  **H 3.26040600 -0.98698900 -1.00209800**  **H 2.50256300 -2.54980100 -1.22531400**  **C 4.06479300 -2.53412900 0.25889800**  **H 3.69928300 -3.41172500 0.79284900**  **H 4.45035300 -1.85208700 1.01733300**  **C 5.19346900 -2.94226900 -0.67592700**  **H 5.59991300 -2.07924400 -1.20097400**  **H 6.00865800 -3.41163700 -0.12894900**  **H 4.84530200 -3.65123600 -1.42542800**  **O 2.87607500 3.17334300 0.71557900**  **H -0.14441900 1.77832400 -0.96001100** |
| **morpholine -IM** | **morpholine -TS2** |
| **O 1.48962200 -0.79552900 -0.15415200**  **C 0.32676400 -1.51077800 -0.02180300**  **N 0.30030500 1.79931600 -0.51380500**  **C -0.45985500 2.81850200 -1.27648800**  **H 0.21465000 3.63936700 -1.49480100**  **H -0.79014300 2.36775500 -2.20519700**  **C 0.66209400 2.26757400 0.84685500**  **H 1.39069100 3.06437500 0.74290300**  **H 1.10736000 1.43332900 1.37435600**  **C 2.69959700 -1.50939900 0.04162300**  **H 2.72184800 -1.92793600 1.04498400**  **H 2.75356800 -2.33852600 -0.65958800**  **C -4.75118000 -1.87411400 0.00410800**  **C -3.78311800 -2.62357200 0.65688000**  **C -4.34628300 -0.77016900 -0.73467200**  **C -3.00768400 -0.43013500 -0.81435600**  **C -2.01280500 -1.17509800 -0.15912100**  **C -2.43924000 -2.29020400 0.58244100**  **H -1.70781500 -2.89048000 1.08955700**  **H -4.07483100 -3.48558400 1.23811800**  **H -5.07655700 -0.17061900 -1.25662800**  **H -2.70416000 0.42302700 -1.40084800**  **N -0.71045100 -0.71562300 -0.26726400**  **O 0.38363700 -2.69960500 0.26503900**  **H -0.22846400 0.86236300 -0.44415200**  **H -5.79330300 -2.14356400 0.06750700**  **C -0.58002000 2.76805800 1.55093200**  **H -0.30586300 3.19436900 2.50937000**  **H -1.27298300 1.94283100 1.72435500**  **C -1.63406500 3.30056700 -0.45049800**  **H -2.35839100 2.49612200 -0.31584200**  **H -2.12256600 4.12084300 -0.96442400**  **C 3.84911300 -0.54932200 -0.16969600**  **H 3.77824600 -0.12538600 -1.17161100**  **H 3.75645200 0.27927200 0.53262100**  **C 5.20341500 -1.22387400 0.00893600**  **H 5.28758800 -2.05546500 -0.69110800**  **H 5.26227600 -1.65632900 1.00808100**  **C 6.36775600 -0.26688500 -0.19812500**  **H 7.32245700 -0.77191100 -0.06582600**  **H 6.35343500 0.15770400 -1.20080200**  **H 6.32831100 0.55864200 0.51093100**  **O -1.20902900 3.78199100 0.80270400**  **H 1.14844600 1.55819300 -1.01528300** | **O 1.56144500 -0.73035200 -0.14048900**  **C 0.42392300 -1.47273000 -0.02561800**  **N 0.12828900 1.75779600 -0.56210600**  **C -0.75820500 2.69235800 -1.28462600**  **H -0.17611200 3.55673300 -1.59207400**  **H -1.13738600 2.19005500 -2.16818600**  **C 0.56446700 2.30081800 0.74115000**  **H 1.22361800 3.14657200 0.56636700**  **H 1.11107700 1.52266400 1.26079600**  **C 2.79160100 -1.42417400 0.01504500**  **H 2.82454800 -1.89395200 0.99463000**  **C -4.64147900 -1.96997600 0.01697200**  **C -3.66138100 -2.64999000 0.72581500**  **C -4.26473900 -0.89206600 -0.77173600**  **C -2.93692400 -0.50750800 -0.84860800**  **C -1.93670100 -1.18164200 -0.13499900**  **C -2.33049700 -2.26840500 0.65864200**  **H -1.58701700 -2.81085500 1.21199600**  **H -3.93344300 -3.49033200 1.34633600**  **H -5.00629000 -0.34930200 -1.33751300**  **H -2.65324700 0.32123800 -1.47760200**  **N -0.63921100 -0.69086500 -0.22889600**  **O 0.49042600 -2.66837100 0.21105300**  **H -0.32964100 0.66652600 -0.42106800**  **H 2.86178600 -2.21202300 -0.73059400**  **H -5.67408500 -2.27442900 0.07600300**  **C -0.64487600 2.74009900 1.53720600**  **H -0.32396400 3.21867000 2.45599000**  **H -1.26178000 1.87669200 1.79465400**  **C -1.89391600 3.12684500 -0.38178500**  **H -2.54787200 2.28252700 -0.16072400**  **H -2.47881200 3.89389000 -0.87768100**  **C 3.91444800 -0.42446900 -0.14635600**  **H 3.82685200 0.05403500 -1.12184400**  **H 3.80490200 0.35957500 0.60282200**  **C 5.28588500 -1.07426000 -0.01244700**  **H 5.35996000 -1.56472100 0.95847000**  **H 5.38829900 -1.85942100 -0.76187600**  **C 6.42428800 -0.07649900 -0.16383500**  **H 6.36661800 0.70245500 0.59487400**  **H 7.39211800 -0.56392100 -0.06550900**  **H 6.39447500 0.40848800 -1.13835200**  **O -1.40604900 3.68183900 0.81746000**  **H 0.94993200 1.57499100 -1.12601800** |
| **morpholine -PC** | |
| **O 1.46643400 -0.94778300 -0.09953200**  **C 0.35185300 -1.67678900 0.03080000**  **N 0.22044500 1.92038300 -0.54998300**  **C -0.34548700 3.03301700 -1.30586700**  **H 0.40215600 3.80686000 -1.50401000**  **H -0.71605600 2.66871200 -2.26045100**  **C 0.66906800 2.36630600 0.76511700**  **H 1.47374900 3.10387800 0.68979300**  **H 1.04213600 1.51147200 1.32206000**  **C 2.70518400 -1.65034800 0.03611400**  **H 2.74071000 -2.12070100 1.01411700**  **H 2.75338500 -2.43111900 -0.71700800**  **C -4.80738600 -1.87724400 -0.04384900**  **C -3.86967200 -2.80971200 0.37012700**  **C -4.37026400 -0.63445200 -0.48016400**  **C -3.02037900 -0.33401600 -0.50422300**  **C -2.07608400 -1.27299400 -0.08540700**  **C -2.51281300 -2.52123400 0.35649600**  **H -1.79885000 -3.25313400 0.68078200**  **H -4.19099600 -3.78030900 0.71400400**  **H -5.08115800 0.10707100 -0.80847000**  **H -2.68785600 0.63079600 -0.85190300**  **N -0.73326400 -0.88215400 -0.12225700**  **O 0.35758500 -2.86652500 0.25117200**  **H -0.54142100 0.10164600 -0.31850100**  **H -5.85898800 -2.11305800 -0.02756100**  **C -0.49934300 2.99012200 1.49855800**  **H -0.17990900 3.39488700 2.45368200**  **H -1.26756400 2.23465200 1.68166000**  **C -1.48198000 3.64481300 -0.51285700**  **H -2.29407300 2.92143800 -0.40777900**  **H -1.86845900 4.52471700 -1.01748900**  **C 3.82316200 -0.64944000 -0.13549300**  **H 3.72154500 0.13150100 0.61736900**  **H 3.72572500 -0.17009000 -1.10916500**  **C 5.19385100 -1.30417600 -0.01645700**  **H 5.28668200 -2.08782500 -0.76850900**  **H 5.27618500 -1.79603600 0.95291900**  **C 6.33179100 -0.30757500 -0.17763000**  **H 6.28276400 0.46992500 0.58304900**  **H 7.29939000 -0.79723200 -0.08968100**  **H 6.29285700 0.17885000 -1.15100900**  **O -1.04607100 4.06224600 0.76205300**  **H 1.00518800 1.53431800 -1.05334200** | |
| **4-methylmorpholine** | **4-methylmorpholine-RC1** |
| **C 0.10337500 -1.19426900 1.16225400**  **H -0.92437200 -1.56247500 1.21158800**  **H 0.65278600 -1.59117200 2.01022600**  **C 0.10337500 -1.19426900 -1.16225400**  **H 0.65278600 -1.59117200 -2.01022600**  **H -0.92437200 -1.56247500 -1.21158800**  **C 0.10337500 0.32012400 -1.18994000**  **H -0.43167700 0.66698700 -2.07146200**  **H 1.13890200 0.67833000 -1.26578300**  **C 0.10337500 0.32012400 1.18994000**  **H -0.43167700 0.66698700 2.07146200**  **H 1.13890200 0.67833000 1.26578300**  **C -0.59007800 2.28274100 0.00000000**  **H -1.12027700 2.63638600 0.88111200**  **H 0.40889800 2.73692900 0.00000000**  **H -1.12027700 2.63638600 -0.88111200**  **N -0.54878200 0.83851600 0.00000000**  **O 0.73266400 -1.68367000 0.00000000** | **O 0.55941900 -0.87681500 -1.42522000**  **N -1.30683500 0.89901500 -0.15890600**  **C -2.50974000 0.57703400 -0.91779000**  **H -3.32458500 1.26497300 -0.66505600**  **H -2.29788400 0.68353200 -1.97903500**  **C -1.54174000 0.68047600 1.26312000**  **H -2.29776300 1.37801900 1.64162700**  **H -0.61782900 0.86043400 1.80734600**  **C 1.63880900 -1.23128600 -0.59845800**  **H 1.28789600 -1.57548500 0.37891600**  **H 2.13100700 -2.08003800 -1.07134000**  **H -0.01415000 -0.25780800 -0.93729600**  **C -2.00800900 -0.74065800 1.50327200**  **H -2.26091400 -0.88250000 2.54921500**  **H -1.21136100 -1.44156300 1.24251500**  **C -0.85793000 2.25138700 -0.42417000**  **H 0.05243300 2.45214600 0.13408600**  **H -0.64375800 2.36395900 -1.48363200**  **H -1.60616300 2.99934000 -0.14201400**  **C -2.93977400 -0.84489100 -0.62277400**  **H -2.17212400 -1.53869100 -0.97185400**  **H -3.87122000 -1.07269100 -1.13113000**  **C 2.63938300 -0.10263600 -0.41289600**  **H 2.13363000 0.74760000 0.04868400**  **H 2.97328600 0.22919200 -1.39663300**  **C 3.83980400 -0.50344500 0.43457300**  **H 4.33785900 -1.35641200 -0.02805500**  **H 3.49504600 -0.84441300 1.41161200**  **C 4.84112400 0.62739800 0.61872600**  **H 4.38094500 1.48315400 1.11059500**  **H 5.68785500 0.31270000 1.22574500**  **H 5.22926500 0.96798900 -0.34007000**  **O -3.16631700 -1.03153600 0.75603300** |
| **4-methylmorpholine-RC2** | **4-methylmorpholine-TS1** |
| **O 1.26498900 -0.60450900 -1.24365700**  **C -1.22151300 -2.11634600 -0.31356000**  **N 1.85260600 2.14130300 -0.66436400**  **C 0.50796700 2.69596400 -0.56463200**  **H 0.53272500 3.78967400 -0.63138200**  **H -0.08961100 2.32299900 -1.39309600**  **C 2.64921000 2.55400500 0.48408700**  **H 2.80304500 3.63925100 0.47839900**  **H 3.62528600 2.07809700 0.42964000**  **C 2.40107500 -1.42967000 -1.28984800**  **H 2.09562000 -2.35224400 -1.77952900**  **C -5.82410500 0.09467100 0.30630000**  **C -5.42263300 -1.17549500 0.69675600**  **C -4.92117900 0.93409400 -0.32903800**  **C -3.62458800 0.51161600 -0.57446800**  **C -3.22878600 -0.76046700 -0.18148700**  **C -4.12961400 -1.60813600 0.45684300**  **H -3.81425400 -2.59344500 0.76003900**  **H -6.11758000 -1.83516300 1.19083200**  **H -5.22386100 1.92198000 -0.63646400**  **H -2.91653300 1.15498300 -1.06974200**  **N -1.91523100 -1.15077200 -0.44385800**  **O -0.48364800 -3.01344600 -0.22904500**  **H 1.53149500 0.28223700 -0.94069400**  **H 3.18096800 -0.98264500 -1.91390000**  **H -6.83205400 0.42596600 0.49567700**  **C 1.95683000 2.15451600 1.77046500**  **H 2.50760800 2.52964700 2.62722700**  **H 1.90562600 1.06560400 1.84188500**  **C 2.48735200 2.50996800 -1.91451100**  **H 3.47630500 2.06288600 -1.96823900**  **H 1.89579300 2.13907800 -2.74708600**  **H 2.59420900 3.59431500 -2.02241100**  **C -0.12531600 2.28596400 0.74876000**  **H -0.25526200 1.20169000 0.77277200**  **H -1.09855400 2.75263200 0.86222900**  **C 2.96717200 -1.74686800 0.08384100**  **H 3.25415300 -0.81454200 0.57402800**  **H 2.17492700 -2.18892500 0.68777700**  **C 4.16686200 -2.68399400 0.03224500**  **H 3.87759700 -3.61180700 -0.46292300**  **H 4.94789000 -2.23903200 -0.58554900**  **C 4.73171200 -3.00138000 1.40907500**  **H 3.98275400 -3.47781300 2.03999000**  **H 5.58504200 -3.67363600 1.34285000**  **H 5.06181600 -2.09594400 1.91630000**  **O 0.65997800 2.70042800 1.84319800** | **O -1.05083500 -0.55532600 0.38752600**  **C 0.52963500 -1.13836600 -0.34698600**  **N -1.04354800 2.10119700 0.83461900**  **C 0.07979900 2.77418500 0.18199500**  **H 0.18193000 3.79363300 0.56569600**  **H 0.99195300 2.23057600 0.40683500**  **C -2.29545800 2.76481500 0.46947600**  **H -2.31231600 3.78216100 0.87169700**  **H -3.12656000 2.21644600 0.90480200**  **C -2.25576000 -1.00434500 -0.22892400**  **H -3.00355600 -0.22630700 -0.10111200**  **H -2.07925200 -1.13721400 -1.29309800**  **C 5.51504100 -0.93374500 -0.06094300**  **C 4.67822900 -1.58539800 -0.95488100**  **C 4.95627900 -0.17530300 0.95827700**  **C 3.58123300 -0.07062500 1.07975900**  **C 2.73421800 -0.72299900 0.18382900**  **C 3.30042400 -1.48586300 -0.83966400**  **H 2.66689800 -1.99899600 -1.54293800**  **H 5.09774700 -2.17872400 -1.75209800**  **H 5.59202800 0.33724800 1.66287600**  **H 3.14289300 0.51472100 1.87185500**  **N 1.35371900 -0.55395000 0.38835500**  **O 0.23622500 -1.85879300 -1.23624300**  **H -1.03457000 0.44915000 0.48110400**  **H 6.58559900 -1.01589400 -0.15690500**  **C -2.44347000 2.81828900 -1.03712800**  **H -3.33171600 3.38170000 -1.30434200**  **H -2.54269900 1.80780300 -1.44013400**  **C -0.86715400 2.06662700 2.27848000**  **H -1.70361500 1.54445400 2.73411400**  **H 0.04743600 1.53221800 2.51785400**  **H -0.80864400 3.07092800 2.70482600**  **C -0.13773500 2.81899100 -1.31543400**  **H -0.13870100 1.80466300 -1.72227100**  **H 0.65999600 3.37831200 -1.79328900**  **C -2.71057000 -2.29967200 0.40595100**  **H -2.86533000 -2.13491400 1.47199900**  **H -1.91414200 -3.03678500 0.30961200**  **C -3.98736500 -2.83456800 -0.22958800**  **H -3.82306700 -2.98563300 -1.29679600**  **H -4.77560000 -2.08605900 -0.14303700**  **C -4.45477700 -4.13797700 0.40060000**  **H -4.65890900 -4.00964700 1.46248400**  **H -5.36614600 -4.49999300 -0.07090900**  **H -3.69838000 -4.91493800 0.30120600**  **O -1.34662500 3.46738100 -1.63625300** |
| **4-methylmorpholine-IM** | **4-methylmorpholine-TS2** |
| **O -1.45057200 -0.85728500 0.14338400**  **C -0.26863500 -1.53476400 -0.00062900**  **N 0.02546500 1.89502200 0.72901500**  **C 1.19109500 2.81542100 0.78872800**  **H 0.82090800 3.78558900 1.10529200**  **H 1.88210300 2.43711100 1.53253900**  **C -0.89365400 2.30689200 -0.36481200**  **H -1.32463700 3.26343800 -0.08586200**  **H -1.67238900 1.55925100 -0.43815600**  **C -2.63244000 -1.60096300 -0.10952000**  **H -2.61119200 -1.98885400 -1.12508100**  **H -2.67995900 -2.45370000 0.56337800**  **C 4.80271100 -1.86578800 -0.01499300**  **C 3.84367400 -2.56712600 -0.73174400**  **C 4.38655300 -0.81741400 0.79440600**  **C 3.04607400 -0.48477200 0.88109700**  **C 2.05960800 -1.18141100 0.16327200**  **C 2.49895600 -2.23999000 -0.65096800**  **H 1.77614200 -2.80334500 -1.20985000**  **H 4.14359100 -3.38519500 -1.36958700**  **H 5.10882900 -0.25577800 1.36721400**  **H 2.73563100 0.32250700 1.52536400**  **N 0.75298600 -0.73066700 0.28198900**  **O -0.29417700 -2.71518100 -0.32731200**  **H 0.36625900 0.89694200 0.52318600**  **H 5.84591100 -2.12970200 -0.08386700**  **C -0.12958400 2.42222600 -1.66629700**  **H -0.79327300 2.79465200 -2.43870000**  **H 0.24094400 1.44206000 -1.97122700**  **C -0.67011700 1.81804100 2.02707000**  **H -1.47522600 1.10064400 1.94061600**  **H 0.03711500 1.48802900 2.77852000**  **H -1.05925600 2.79575000 2.28665600**  **C 1.85297300 2.91516500 -0.56942800**  **H 2.29469400 1.95708900 -0.84684100**  **H 2.64311500 3.65664100 -0.52566000**  **C -3.82259500 -0.68980400 0.09323900**  **H -3.80476400 -0.29779100 1.11024800**  **H -3.73891900 0.16489500 -0.57819800**  **C -5.14292300 -1.40847100 -0.15622100**  **H -5.21885500 -2.26632900 0.51239400**  **H -5.15018200 -1.80812900 -1.17063400**  **C -6.35019900 -0.50393000 0.04025200**  **H -7.27929900 -1.03970100 -0.14350000**  **H -6.38778200 -0.11405100 1.05634000**  **H -6.31829900 0.34667800 -0.63895700**  **O 0.93733100 3.33258200 -1.55274300** | **O -1.52619400 -0.78237700 0.13906400**  **C -0.36129800 -1.47443400 0.00981300**  **N 0.18419900 1.82941300 0.74449600**  **C 1.41107300 2.65499600 0.74934800**  **H 1.14757600 3.65359400 1.09147900**  **H 2.11549300 2.22015300 1.44908300**  **C -0.75233500 2.33194900 -0.28435900**  **H -1.09332700 3.32024100 0.01658000**  **H -1.59752400 1.65659400 -0.32607900**  **C -2.72160900 -1.51700600 -0.08975700**  **H -2.70281500 -1.93942400 -1.09095800**  **C 4.68123500 -1.94950900 -0.03880000**  **C 3.72898800 -2.45595700 -0.91297400**  **C 4.28011900 -1.04646500 0.93452600**  **C 2.95223500 -0.66267200 1.03246800**  **C 1.98210600 -1.16191400 0.15614400**  **C 2.40124400 -2.07043100 -0.82483400**  **H 1.67795900 -2.47456400 -1.50926400**  **H 4.02231300 -3.15700900 -1.67942300**  **H 5.00062300 -0.64237100 1.62879600**  **H 2.64716200 0.02380400 1.80609600**  **N 0.67875000 -0.67840500 0.27267400**  **O -0.37921000 -2.65880200 -0.28657800**  **H 0.44567200 0.68192800 0.50557200**  **H -2.78120800 -2.34301200 0.61427400**  **H 5.71289000 -2.25355000 -0.11475400**  **C -0.06169800 2.40414700 -1.62981100**  **H -0.73455800 2.84278900 -2.35877900**  **H 0.20750800 1.40203400 -1.96852100**  **C -0.44259200 1.80290300 2.07133500**  **H -1.31040000 1.15586100 2.03365900**  **H 0.26939500 1.40844900 2.78793000**  **H -0.74037500 2.80453600 2.36780800**  **C 2.01104500 2.72159500 -0.63960300**  **H 2.36109300 1.73540900 -0.94847300**  **H 2.85865200 3.39848600 -0.63355900**  **C -3.89390600 -0.57711200 0.07898700**  **H -3.86731500 -0.14787200 1.08047800**  **H -3.79379500 0.24951100 -0.62438200**  **C -5.22727600 -1.28034800 -0.14310000**  **H -5.24234300 -1.71899500 -1.14114000**  **H -5.31944700 -2.10964600 0.55860700**  **C -6.41663600 -0.34542300 0.01719400**  **H -6.36806000 0.47695100 -0.69490600**  **H -7.35593300 -0.86994300 -0.14584300**  **H -6.44624500 0.08459500 1.01720100**  **O 1.08317800 3.22098800 -1.57262600** |
| **4-methylmorpholine-PC** | |
| **O -1.18494700 0.26369300 -1.16840300**  **C -0.08900600 0.66529400 -0.52268000**  **C -2.36698100 1.04992600 -1.00394400**  **H -2.10847300 2.10199300 -1.03518900**  **C 4.87475000 -0.29767300 0.61191900**  **C 4.04585700 0.74777000 0.98579700**  **C 4.36890300 -1.29680900 -0.20784700**  **C 3.05743400 -1.24882800 -0.64374100**  **C 2.22493600 -0.19535700 -0.26496800**  **C 2.72787400 0.81059400 0.55709600**  **H 2.09640000 1.62535200 0.85385100**  **H 4.42265200 1.53287300 1.62212600**  **H 4.99553300 -2.12016100 -0.51140200**  **H 2.66946600 -2.02869300 -1.28066600**  **N 0.91108000 -0.22260900 -0.74714700**  **O -0.01882600 1.67093200 0.14470500**  **H -2.96901500 0.81684300 -1.87267200**  **H 5.89670100 -0.33465400 0.95220600**  **C -3.08848200 0.74700300 0.29196900**  **H -2.43774000 1.02392700 1.11599900**  **H -3.96311400 1.41128600 0.33631100**  **N -3.45810000 -0.64545100 0.45938900**  **C -3.87099900 -0.90213800 1.82321800**  **H -4.76661500 -0.33686700 2.11222700**  **H -4.08925100 -1.95982100 1.94784700**  **H -3.07062100 -0.63771700 2.50958200**  **C -4.48557500 -1.06618500 -0.46991700**  **H -4.72067000 -2.11350400 -0.29946000**  **H -5.41339700 -0.48867400 -0.36470100**  **H -4.14279800 -0.96948700 -1.49554300**  **H 0.66800100 -1.00486400 -1.32803700** | |
